# Molecular Characterization and Antifungal Susceptibility of Clinical *Fusarium* Species From Brazil

**DOI:** 10.3389/fmicb.2019.00737

**Published:** 2019-04-10

**Authors:** Patricia F. Herkert, Abdullah M. S. Al-Hatmi, Gabriel L. de Oliveira Salvador, Marisol D. Muro, Rosângela L. Pinheiro, Márcio Nucci, Flávio Queiroz-Telles, G. Sybren de Hoog, Jacques F. Meis

**Affiliations:** ^1^Instituto Carlos Chagas, Fundação Oswaldo Cruz, Curitiba, Brazil; ^2^Instituto Nacional de Ciência e Tecnologia de Inovação em Doenças de Populações Negligenciadas, Brasília, Brazil; ^3^Centre of Expertise in Mycology Radboudumc/CWZ, Nijmegen, Netherlands; ^4^Department of Medical Mycology, Westerdijk Fungal Biodiversity Institute, Utrecht, Netherlands; ^5^Directorate General of Health Services, Ministry of Health, Ibri Hospital, Ibri, Oman; ^6^Department of Internal Medicine, Federal University of Paraná, Curitiba, Brazil; ^7^Laboratory of Mycology, Hospital de Clínicas, Federal University of Paraná, Curitiba, Brazil; ^8^Department of Internal Medicine, Hematology Service, University Hospital, Federal University of Rio de Janeiro, Rio de Janeiro, Brazil; ^9^Infectious Diseases Unit, Department of Public Health, Hospital de Clínicas, Federal University of Paraná, Curitiba, Brazil; ^10^Postgraduate Program in Microbiology, Parasitology and Pathology, Biological Sciences, Department of Basic Pathology, Federal University of Paraná, Curitiba, Brazil; ^11^Department of Medical Microbiology and Infectious Diseases, Canisius-Wilhelmina Hospital, Nijmegen, Netherlands

**Keywords:** fusariosis, antifungal, fungicide, susceptibility, *Fusarium*, molecular identification

## Abstract

*Fusarium* is widely distributed in the environment and is involved with plant and animal diseases. In humans, several species and species complexes (SC) are related to fusariosis, i.e., *F. solani* SC, *F. oxysporum* SC, *F. fujikuroi* SC, *F. dimerum, F. chlamydosporum, F. incarnatum-equiseti*, and *F. sporotrichoides*. We aimed to investigate the susceptibility of *Fusarium* clinical isolates to antifungals and azole fungicides and identify the species. Forty-three clinical *Fusarium* isolates were identified by sequencing translation elongation factor 1-alpha (*TEF1*α) gene. Antifungal susceptibility testing was performed to the antifungals amphotericin B, itraconazole, voriconazole, posaconazole, and isavuconazole, and the azole fungicides difenoconazole, tebuconazole, and propiconazole. The isolates were recovered from patients with median age of 36 years (range 2–78 years) of which 21 were female. Disseminated fusariosis was the most frequent clinical form (*n* = 16, 37.2%) and acute lymphoblastic leukemia (*n* = 7; 16.3%) was the most commonly underlying condition. A few species described in *Fusarium solani* SC have recently been renamed in the genus *Neocosmospora*, but consistent naming is yet not possible. *Fusarium keratoplasticum* FSSC 2 (*n* = 12) was the prevalent species, followed by *F. petroliphilum* FSSC 1 (*n* = 10), *N. gamsii* FSSC 7 (*n* = 5), *N. suttoniana* FSSC 20 (*n* = 3), *F. solani sensu stricto* FSSC 5 (*n* = 2), *Fusarium* sp. FSSC 25 (*n* = 2), *Fusarium* sp. FSSC 35 (*n* = 1), *Fusarium* sp. FSSC18 (*n* = 1), *F. falciforme* FSSC 3+4 (*n* = 1), *F. pseudensiforme* (*n* = 1), and *F. solani* f. *xanthoxyli* (*n* = 1). Amphotericin B had activity against most isolates although MICs ranged from 0.5 to 32 μg mL^-1^. *Fusarium keratoplasticum* showed high MIC values (8–>32 μg mL^-1^) for itraconazole, voriconazole, posaconazole, and isavuconazole. Among agricultural fungicides, difenoconazole had the lowest activity against FSSC with MICs of >32 μg mL^-1^ for all isolates.

## Introduction

The fungal genus *Fusarium* is widely distributed as saprobes in the environment but is also able to cause cross-kingdom disease in both plants and mammals (Gauthier and Keller, 2013; van Diepeningen and de Hoog, 2016). In humans, the disease may manifest in different ways, depending on the portal of entry and the host’s immune status. Invasive fusariosis is the most severe manifestation that predominantly affects immunocompromised hosts with hematological malignancies, neutropenia, or glucocorticoid exposure (Nucci et al., 2003, 2004, 2019; de Souza et al., 2014). In immunocompetent hosts, the fungus may cause onychomycosis (Guevara-Suarez et al., 2016), keratitis (Tupaki-Sreepurna et al., 2017a) or other (sub)cutaneous disorders. The most frequent fungal diseases caused by *Fusarium* species are onychomycosis and keratitis, although other clinical presentations are also observed, such as fungemia, mycetoma, skin infection, lung disease (including allergic disease, hypersensitivity pneumonitis, colonization of a pre-existing cavity, pneumonia in severely immunocompromised patients), and other rare infections (endocarditis, urinary tract infection, osteomyelitis, etc.) (Sierra-Hoffman et al., 2005; Su et al., 2007; Nucci et al., 2015; Kassar et al., 2016).

Species belonging to *Fusarium* are distributed into several species complexes (SC), some of which are important in human and veterinary mycology, particularly *F. solani* SC, *F. oxysporum* SC, *F. fujikuroi* SC, *F. dimerum, F. chlamydosporum, F. incarnatum-equiseti*, and *F. sporotrichoides* (van Diepeningen et al., 2014; Salah et al., 2015; Al-Hatmi et al., 2016a; Hassan et al., 2016). *Fusarium graminearum, F. culmorum, F. fujikuroi* SC, *F. solani* SC, and *F. oxysporum* SC may additionally be found as plant pathogens in maize, wheat, rice, soybean, and tomato crops (Basler, 2016; Costa et al., 2016; Kim et al., 2016; Manzo et al., 2016). Some *Fusarium* species produce mycotoxins during growth in plant tissue, which may contaminate cereal grains and derivatives, making them unsuitable for consumption and causing great agricultural losses (Milicevic et al., 2010; Sobrova et al., 2010).

In attempts to reduce agricultural losses caused by fungal diseases, many strategies have been used, including augmentation of plant resistance, spraying of chemicals, biological control, integrated disease management (Singh et al., 2016), and fungicide use, especially azoles (Hof, 2001). The continuing uncontrolled use of fungicides may lead to selective pressure on environmental fungi (Deising et al., 2008). Due to the structural similarity of azoles used in agriculture and medicine, cross-resistance may be observed in clinical fungi (Meis et al., 2016; Verweij et al., 2016). Studies have been performed to test the hypothesis whether fungicide use in agroecosystems may lead to antifungal resistance in *Aspergillus fumigatus* in the clinic (Snelders et al., 2008; Chowdhary et al., 2012, 2013; Meis et al., 2016; Alvarez-Moreno et al., 2017).

In the medical field, amphotericin B, voriconazole, and posaconazole are the main antifungal drugs recommended for prophylaxis and treatment of human fusariosis (Lortholary et al., 2010; Tortorano et al., 2014; Clark et al., 2015; Nucci et al., 2015; Taj-Aldeen et al., 2016; Al-Hatmi et al., 2018b). Most *Fusarium* species exhibit high minimal inhibitory concentrations (MICs) to currently used antifungals, especially azoles (Katiyar and Edlind, 2009; Fan et al., 2013; Al-Hatmi et al., 2015).

Here we aimed to investigate the susceptibility of *Fusarium* clinical isolates to commonly used antifungals and fungicides and identify the species. For this study, we used strains that were isolated from patients with fusariosis diagnosed in two tertiary Brazilian hospitals in southern Brazil.

## Materials and Methods

### Strains and Clinical Data

Forty-three clinical *Fusarium* isolates were available from the Laboratory of Mycology at the Federal University of Paraná Hospital, Curitiba, Brazil and Federal University of Rio de Janeiro Hospital, Rio de Janeiro, Brazil, recovered from 40 patients cared between 1985 and 2015. Three patients (32, 36, and 38) had each two isolates recovered, as specified in the Table 1. The patient’s medical records were reviewed to collect minimal clinical information such as age, gender, treatment, and outcome.

**Table 1:** *Fusarium* isolates data.

Isolate no.	Species complex	Species	Patient	Type of fusariosis	Underlying disease	Source	Treatment	GenBank accession no.
Fu02	FSSC25	*Fusarium* sp.	1	Disseminated	Unknown	Blood	VOR	MG738163
Fu14	FSSC2	*F. keratoplasticum*	2	Disseminated	AML	Skin	VOR	MG738189
Fu27	FSSC2	*F. keratoplasticum*	3	Cutaneous	Arterial insufficiency on legs	Skin	VOR	MG738193
Fu34	FSSC5	*F. solani s.s.*	4	Keratitis	None	Eye	VOR	MG738195
Fu37	FSSC2	*F. keratoplasticum*	5	Cutaneous	None	Skin	VOR	MG738184
Fu50	FSSC1	*F. petroliphilum*	6	Disseminated	Myelodysplasia	Skin	AMB	MG738167
Fu51	FSSC1	*F. petroliphilum*	7	Disseminated	AML	Blood	FLU	MG738168
Fu56	FFSC	*F. napiforme*	8	Cutaneous	Fanconi anemia	Blood	VOR + AMB	MG738202
Fu66	FSSC3+4	*F. falciforme*	9	Keratitis	None	Eye	VOR	MG738197
Fu71	FFSC	*F. verticillioides*	10	Disseminated	AML	Skin	VOR	MG738201
Fu72	FSSC7	*N. gamsii*	11	Cutaneous	ALL	Blood	VOR	MG738177
Fu73	FSSC7	*N. gamsii*	12	Disseminated	Non-Hodgkin lymphoma	Skin	VOR + AMB	MG738178
Fu75	FSSC1	*F. petroliphilum*	13	Keratitis	None	Eye	not done	MG738169
Fu77	FSSC2	*F. keratoplasticum*	14	Disseminated	Purpura amegakaryocytic	Skin	not done	MG738190
Fu78	FFSC	*F. subglutinans*	15	Disseminated	Aplastic anemia	Blood	VOR + AMB	MG738203
Fu80	FSSC7	*N. gamsii*	16	Unknown	Unknown	Skin	Unknown	MG738179
Fu86	FSSC25	*Fusarium* sp.	17	Unknown	Unknown	Skin	Unknown	MG738164
Fu87	FSSC	*Fusarium* sp.	18	Cutaneous	ALL	Blood	VOR	MG738166
Fu89	FSSC35	*Fusarium* sp.	19	Disseminated	Unknown	Blood	VOR	MG738162
Fu92	FSSC1	*F. petroliphilum*	20	Cutaneous	Aplastic anemia	Skin	VOR + AMB	MG738170
Fu93	FSSC20	*N. suttoniana*	21	Disseminated	CML	Skin	VOR + AMB	MG738198
Fu94	FSSC	*F. xanthoxyli*	22	Disseminated	Unknown	Skin	VOR	MG738182
Fu96	FSSC2	*F. keratoplasticum*	23	Disseminated	ALL	Skin	VOR	MG738185
Fu97	FSSC2	*F. keratoplasticum*	24	Disseminated	ALL	Endotracheal aspirate	VOR + AMB	MG738194
Fu99	FSSC1	*F. petroliphilum*	25	Cutaneous	Aplastic anemia	Skin	VOR + ISA	MG738171
Fu100	FSSC20	*N. suttoniana*	26	Keratitis	None	Eye	VOR	MG738199
Fu101	FSSC2	*F. keratoplasticum*	27	Disseminated	Myocardium revascularization	Skin	VOR	MG738183
Fu103	FSSC20	*N. suttoniana*	28	Keratitis	None	Eye	VOR	MG738200
Fu105	FSSC2	*F. keratoplasticum*	29	Disseminated	Myelodysplasia	Skin	VOR	MG738191
Fudm2	FSSC7	*N. gamsii*	30	Disseminated	ALL	Blood	VOR + AMB	MG738180
FuB302.1	FSSC2	*F. keratoplasticum*	31	Unknown	Rheumatoid arthritis	Skin	VOR	MG738192
FuB371	FSSC5	*F. solani s.s.*	32	Unknown	ALL	Skin	VOR	MG738196
FuB391	FSSC33	*F. pseudensiforme*	33	Unknown	Unknown	Skin	Unknown	MG738161
FuB478	FSSC2	*F. keratoplasticum*	34	Unknown	AML	Skin	AMB	MG738186
FuB560	FSSC7	*N. gamsii*	35	Unknown	CML	Skin	VOR + lipid AMB	MG738181
FuB604	FSSC1	*F. petroliphilum*	36	Unknown	ALL	Synovial fluid	VOR	MG738172
FuB665	FSSC1	*F. petroliphilum*	36	Unknown	ALL	Synovial fluid	VOR	MG738173
FuB817	FSSC1	*F. petroliphilum*	37	Unknown	Myelodysplasia	Skin	VOR	MG738174
FuB920	FSSC1	*F. petroliphilum*	32	Unknown	ALL	Synovial fluid	VOR	MG738175
FuB935	FSSC2	*F. keratoplasticum*	38	Unknown	AML	Skin	VOR + lipid AMB	MG738187
FuB936	FSSC2	*F. keratoplasticum*	38	Unknown	AML	Skin	VOR + lipid AMB	MG738188
FuH79A	FSSC18	*Fusarium* sp.	39	Unknown	AML	Blood	VOR + lipid AMB	MG738165
FuH05	FSSC1	*F. petroliphilum*	40	Unknown	Unknown	Blood	Unknown	MG738176


*FSSC, *Fusarium solani* species complex; FFSC, *Fusarium fujikuroi* species complex; *s.s*., *sensu stricto*; ALL, acute lymphoblastic leukemia; AML, acute myeloid leukemia; CML, chronic myeloid leukemia; AMB, amphotericin B; FLU, fluconazole; ISA, isavuconazole; VOR, voriconazole; Unknown, no data available.*

### DNA Isolation, PCR, and Sequencing

*Fusarium* isolates were cultured on Sabouraud dextrose agar plus chloramphenicol (SDA; Difco Laboratories, Detroit, MI, United States). Culture plates were incubated at 26 and 37°C and observed daily for growth up to 7 days. Initial identification of *Fusarium* isolates was based on macroscopic colony morphology and microscopic features in a lacto-phenol wet mount preparation according to standard laboratory procedures. Final identification was done using molecular methods. DNA extraction was performed as described by Khodavaisy et al. (2016). Conidia were suspended in 400 μL bacterial lysis buffer (Roche Diagnostics, Almere, Netherlands) followed by mechanical lysis in a MagNA Lyser (Roche Diagnostics) for 30 s at 4,500 × *g*. Cells were inactivated for 10 min by heating at 100°C and 200 μL of the solution was used for automated DNA extraction by using the MagNA Pure 96 platform (Roche Diagnostics) with a final elution volume of 100 μL.

Fragments of the translation elongation factor 1-alpha (*TEF1*α) gene were amplified and sequenced using PCR protocols following the methods published by Al-Hatmi et al. (2014) with primers EF1 (5′-ATGGGTAAGGA(A/G)GACAAGAC-3′) and EF2 (5′-GGA(G/A)GTACCAGT(G/C)ATCATGTT-3′) (O’Donnell et al., 1998). Sequencing reaction mixtures contained 1 ng/μL of template DNA, 1 pmol/μL, 0.7 μL of BigDye^TM^ terminator (Applied Biosystems, Foster City, CA, United States), 3 μL buffer and ultra-pure water to 10 μL final volume. Sequencing PCR was performed as follows: 95°C for 1 min, followed by 30 cycles consisting of 95°C for 10 s, 50°C for 5 s and 60°C for 2 min. Sequencing was done on an ABI 3730xL automatic sequencer (Applied Biosystems).

### Alignment and Phylogenetic Analyses

For preliminary identification, a homology search for the sequences of *TEF1*α was done using the BLAST tool in NCBI database, the CBS database, FUSARIUM-ID (Geiser et al., 2004) and the *Fusarium* MLST (O’Donnell et al., 2010) database down to species and haplotype level. DNA sequences were edited, and consensus sequences were assembled by the SeqMan package of Lasergene software (DNAStar, Madison, WI, United States). Retrieved alignments were manually corrected to avoid mis-paired bases. Sequences were exported as FASTA files. Sequences of *TEF1*α were aligned with MAFFT program^1^ and adjusted in MEGA6 (Tamura et al., 2013). The best-fit model of evolution was determined by MEGA6. Maximum likelihood (ML) analysis was done with RAxML-VI-HPC v. 7.0.3 with non-parametric bootstrapping using 1000 replicates. GenBank accession numbers are shown in Table 1.

### Antifungal Susceptibility Testing

Antifungal susceptibility testing by the broth microdilution method was performed according to the CLSI protocol M38-A2 (Clinical and Laboratory Standards Institute [CLSI], 2008). Antifungal agents tested were amphotericin B (Bristol Myers Squibb, Woerden, Netherlands), itraconazole (Janssen Pharmaceutica, Beerse, Belgium), voriconazole (Pfizer, Sandwich, United Kingdom), posaconazole (Merck, NJ, United States) and isavuconazole (Basilea Pharmaceutica, Basel, Switzerland). The fungicides used were difenoconazole, tebuconazole and propiconazole (all from Sigma-Aldrich, St. Louis, MO, United States). The concentrations of antifungals ranged from 0.031 to 32 μg mL^-1^. *Fusarium* isolates were cultured onto Sabouraud glucose agar until sporulation at 30°C and the inocula were adjusted to 1.8–3 × 10^6^ CFU/mL in saline supplemented with 0.05% Tween 20 to perform the test. Microdilution plates were incubated at 35°C for 48 h and the MICs were defined as the lowest concentration able to complete growth inhibition when compared with the drug free growth control. *Aspergillus flavus* ATCC 204304, *Candida parapsilosis* ATCC 22019 and *C. krusei* ATCC 6258 reference strains were used as quality controls (Clinical and Laboratory Standards Institute [CLSI], 2008). Interpretation of the MIC values was based on Epidemiological Cutoff Values (ECV) according to previous literature data (Espinel-Ingroff et al., 2016). MIC_50_ and MIC_90_ were obtained by ordering the data for each antifungal in ascending order and selecting the median and 90th quantile, respectively. Geometric mean MICs were calculated using Microsoft Office Excel 2010 software (Microsoft, Redmond, WA, United States). When the MIC was more or less than dilutions tested, 1 log_2_ dilution higher or 1 log_2_ dilution lower was considered for calculating the geometric mean.

## Results

### Clinical Data

The median age of the 40 patients was 36 years (range 2–78 years) and 21 were female. Disseminated fusariosis was the most frequent clinical form (*n* = 16, 37.2%), followed by cutaneous infections (*n* = 7; 16.3%) and keratitis (*n* = 5; 11.6%). *Fusarium* strains were isolated most frequently from the skin (*n* = 24; 55.8%), blood (*n* = 10; 23.2%), and eye (*n* = 5; 11.6%). Acute lymphoblastic leukemia (*n* = 7; 16.3%) and acute myeloid leukemia (*n* = 6; 13.9%) were the most commonly underlying conditions. Twelve out of 16 cases of disseminated fusariosis occurred in patients with hematological malignancies. Voriconazole monotherapy was the treatment in 21 (48.8%) patients, 13 of which (61.9%) had a favorable response to therapy. Combination therapy with voriconazole and deoxycholate amphotericin B was given to 7 (16.3%) patients, and voriconazole plus liposomal amphotericin B in 3 patients (7%). Other therapies were deoxycholate amphotericin B alone (*n* = 2; 4.7%), fluconazole alone (*n* = 1; 2.3%), and voriconazole associated with itraconazole (*n* = 1; 2.3%). For 2 (4.7%) patients no therapy was given. Information about treatment was not available in 6 cases. The isolates and respective patients’ clinical data are shown in Table 1.

### Molecular Identification and Phylogeny

**FIGURE 1:**
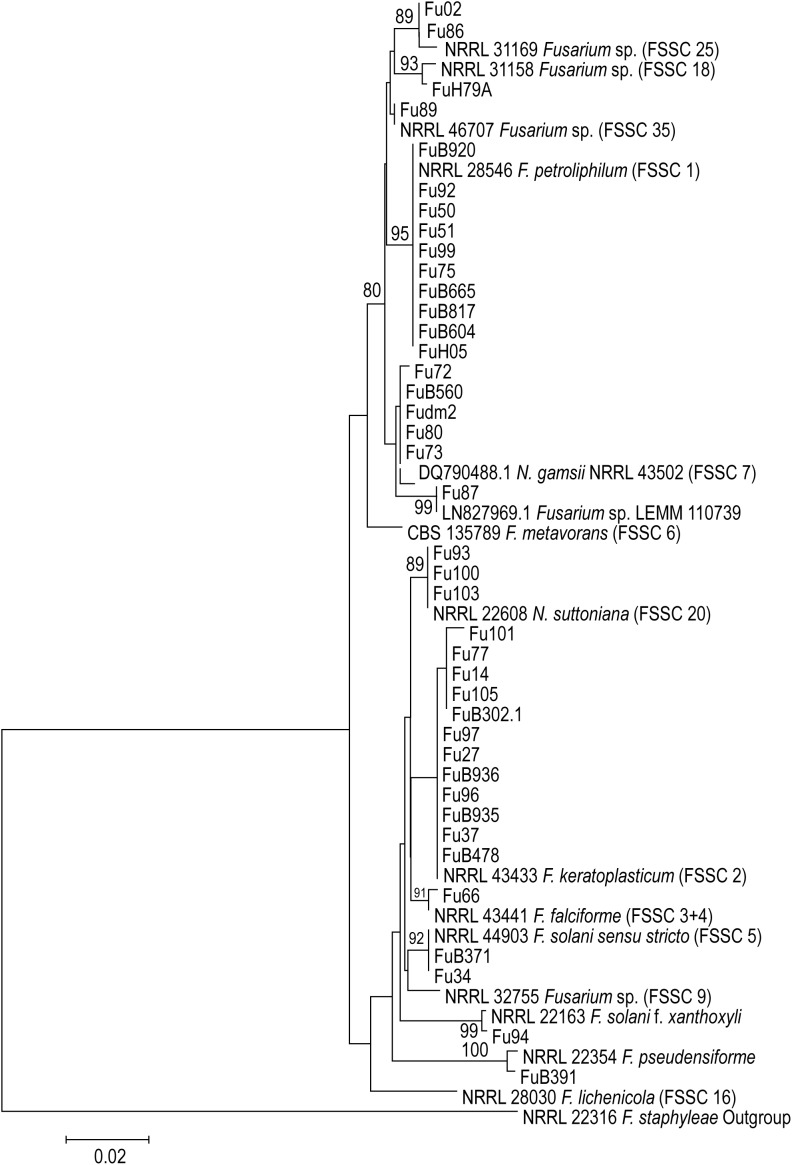
Maximum likelihood (ML) phylogenetic tree created from TEF1 sequences of 55 *Fusarium* sequences. Total alignment length is 707 bp. Thousand bootstrap-replications. The tree was rooted with NRRL 22316 *F. staphyleae*.

Phylogenetic analysis based on *TEF1*α sequences was conducted in order to position the isolates in the *Fusarium solani* complex and their respective species complexes (Figure 1). The analysis included 55 sequences from different species, and one outgroup taxa (NRRL 22316 *F. staphyleae*). Within FSSC, *F. keratoplasticum* FSSC 2 (*n* = 12) was most often involved in cases of fusariosis, followed by *F. petroliphilum* FSSC 1 (*n* = 10), *Neocosmospora gamsii* FSSC 7 (*n* = 5), *N. suttoniana* FSSC 20 (*n* = 3), *F. solani sensu stricto* FSSC 5 (*n* = 2), *Fusarium* sp. FSSC 25 (*n* = 2), *Fusarium* sp. FSSC 35 (*n* = 1), *Fusarium* sp. FSSC 18 (*n* = 1), *F. falciforme* FSSC 3+4 (*n* = 1), *F. pseudensiforme* (*n* = 1), and *F. solani* f. *xanthoxyli* (*n* = 1). One isolate clustered in a separate clade (unknown species/haplotype) forming a distinct, well-supported, unnamed lineage and which matched only with a single strain from Colombia (LEMM 110739, GenBank accession no. LN827969, misidentified as *Fusarium solani*). We also identified the following members of the *Fusarium fujikuroi* species complex (FFSC): *F. subglutinans* (*n* = 1), *F. verticillioides* (*n* = 1), and *F. napiforme* (*n* = 1) which are not included in the phylogenetic analysis.

### Antifungal Susceptibility Profiles

MICs are shown in Tables 2, 3. Amphotericin B had relatively high activity with MICs ranging from 0.5 to 32 μg mL^-1^, except for the isolates Fu73 (novel lineage) and Fu80 (*Neocosmospora gamsii* FSSC7), which showed MIC values of 8 and 32 μg mL^-1^, respectively. All isolates exhibited high MICs to itraconazole with MICs >32 μg mL^-1^. The FSSC had MIC values of posaconazole and difenoconazole higher than 32 μg mL^-1^. Other azoles showed to be less effective against FSSC isolates with high MIC values of 8–>32 μg mL^-1^. *Fusarium keratoplasticum* showed high MIC values (8–>32 μg mL^-1^) for itraconazole, voriconazole, posaconazole and isavuconazole. In counterpart, azoles showed activity against FFSC with MIC values ranges of 1–8 μg mL^-1^ and with only one isolate of *F. napiforme* showing MIC of >32 μg mL^-1^ for posaconazole.

**Table 2:** Minimal inhibitory concentrations of *Fusarium* clinical isolates.

Species complex	Antifungal	No. of isolates per MIC value (μg mL^-1^)											
		
		0.031	0.062	0.125	0.25	0.5	1	2	4	8	16	32	>32
FSSC (*n* = 40)	**Amphotericin B**					10	**16**	9	4			1	
	**Itraconazole**												**40**
	**Voriconazole**							1	2	**21**	7	3	6
	**Posaconazole**												**40**
	**Isavuconazole**											10	**30**
	**Difenoconazole**												**40**
	**Tebuconazole**										3	4	**33**
	**Propiconazole**										1		**39**
FFSC (*n* = 3)	**Amphotericin B**						1	2					
	**Itraconazole**												3
	**Voriconazole**							2	1				
	**Posaconazole**					1	1						1
	**Isavuconazole**								3				
	**Difenoconazole**								1	2			
	**Tebuconazole**							1	2				
	**Propiconazole**							1		2			


*FSSC, *Fusarium solani* species complex; FFSC, *Fusarium fujikuroi* species complex; MIC, minimum inhibitory concentration. The modes are depicted in bold.*

**Table 3:** Individual minimal inhibitory concentration (μg mL^-1^) of all *Fusarium* spp. and *Neocosmospora* spp. isolates.

Isolate	Identification – EF	Minimal inhibitory concentration (μg mL^-1^)							
		**AMB**	**ITC**	**VOR**	**POS**	**ISA**	**DIF**	**TEB**	**PRO**

Fu101	*F. keratoplasticum* (FSSC 2)	4	64	16	64	64	64	64	64
Fu77	*F. keratoplasticum* (FSSC 2)	2	64	16	64	64	64	64	64
Fu14	*F. keratoplasticum* (FSSC 2)	0.5	64	64	64	64	64	64	64
Fu105	*F. keratoplasticum* (FSSC 2)	2	64	8	64	64	64	64	64
FuB302.1	*F. keratoplasticum* (FSSC 2)	2	64	16	64	64	64	64	64
Fu97	*F. keratoplasticum* (FSSC 2)	1	64	16	64	64	64	64	64
Fu27	*F. keratoplasticum* (FSSC 2)	4	64	64	64	64	64	64	64
FuB936	*F. keratoplasticum* (FSSC 2)	1	64	8	64	64	64	64	64
Fu96	*F. keratoplasticum* (FSSC 2)	1	64	8	64	32	64	16	64
FuB935	*F. keratoplasticum* (FSSC 2)	2	64	8	64	64	64	64	64
Fu37	*F. keratoplasticum* (FSSC 2)	1	64	32	64	64	64	64	64
FuB478	*F. keratoplasticum* (FSSC 2)	2	64	8	64	64	64	64	64
	Range^∗^	0.5–4	64	8–64	64	32–64	64	16–64	64
	MIC_50_^∗^	2	64	16	64	64	64	64	64
	MIC_90_^∗^	4	64	64	64	64	64	64	64
	Geometric mean^∗^	1.58	64	16	64	60.4	64	57.01	64
FuB920	*F. petroliphilum* (FSSC 1)	1	64	8	64	64	64	64	64
Fu92	*F. petroliphilum* (FSSC 1)	1	64	8	64	32	64	64	64
Fu50	*F. petroliphilum* (FSSC 1)	2	64	4	64	32	64	64	64
Fu51	*F. petroliphilum* (FSSC 1)	0.5	64	16	64	64	64	64	64
Fu99	*F. petroliphilum* (FSSC 1)	1	64	8	64	32	64	32	64
Fu75	*F. petroliphilum* (FSSC 1)	0.5	64	8	64	64	64	64	64
FuB665	*F. petroliphilum* (FSSC 1)	1	64	8	64	64	64	64	64
FuB817	*F. petroliphilum* (FSSC 1)	0.5	64	8	64	32	64	64	64
FuB604	*F. petroliphilum* (FSSC 1)	1	64	8	64	32	64	64	64
FuH05	*F. petroliphilum* (FSSC 1)	0.5	64	8	64	64	64	64	64
	Range^∗^	0.5–2	64	4–16	64	32–64	64	32–64	64
	MIC_50_^∗^	1	64	8	64	32	64	64	64
	MIC_90_^∗^	1	64	8	64	64	64	64	64
	Geometric mean^∗^	0.81	64	8	64	45.25	64	59.71	64
Fu72	*N. gamsii* (FSSC 7)	1	64	64	64	64	64	64	64
FuB560	*N. gamsii* (FSSC 7)	2	64	8	64	32	64	32	64
Fudm02	*N. gamsii* (FSSC 7)	4	64	8	64	64	64	64	64
Fu80	*N. gamsii* (FSSC 7)	32	64	8	64	64	64	64	64
Fu73	*N. gamsii* (FSSC 7)	4	64	8	64	64	64	64	64
Fu93	*N. suttoniana* (FSSC 20)	1	64	64	64	64	64	64	64
Fu100	*N. suttoniana* (FSSC 20)	0.5	64	64	64	64	64	64	64
Fu103	*N. suttoniana* (FSSC 20)	1	64	32	64	64	64	64	64
Fu02	*Fusarium* sp. (FSSC 25)	0.5	64	32	64	64	64	32	64
Fu86	*Fusarium* sp. (FSSC 25)	1	64	8	64	64	64	64	64
FuB371	*F. solani sensu stricto* (FSSC 5)	2	64	8	64	64	64	64	64
Fu34	*F. solani sensu stricto* (FSSC 5)	0.5	64	16	64	64	64	64	64
FuH79A	*Fusarium* sp. (FSSC 18)	2	64	8	64	64	64	32	64
Fu89	*Fusarium* sp. (FSSC 35)	0.5	64	16	64	64	64	64	64
Fu87	*Fusarium* sp.	1	64	4	64	32	64	64	64
Fu66	*F. falciforme* (FSSC 3+4)	0.5	64	2	64	32	64	16	16
Fu94	*F. solani* f. *xanthoxyli*	1	64	64	64	64	64	64	64
FuB391	*F. pseudensiforme*	1	64	8	64	32	64	16	64
Fu78	*F. subglutinans*	1	64	2	0.5	4	8	4	2
Fu71	*F. verticillioides*	2	64	4	1	4	8	4	8
Fu56	*F. napiforme*	2	64	2	64	4	4	2	8


*FSSC, *Fusarium solani* species complex; FFSC, *Fusarium fujikuroi* species complex; AMB, amphotericin B; ITC, itraconazole; VOR, voriconazole; POS, posaconazole; ISA, isavuconazole; DIF, difenoconazole; TEB, tebuconazole; PRO, propiconazole. ^∗^Values calculated for species with sufficient number of isolates.*

Among the agricultural fungicides, difenoconazole had the lowest activity against FSSC with MICs of >32 μg mL^-1^ for all isolates, followed by propiconazole and tebuconazole. In contrast, the three fungicides showed activity against FFSC, with MIC ranges of 2–8 μg mL^-1^.

## Discussion

Invasive fusariosis is a severe disease that affects immunocompromised patients, mostly those with underlying hematological malignancies (Nucci et al., 2003, 2013; Nucci and Anaissie, 2007; Campo et al., 2010; Carlesse et al., 2017). In agreement with the literature, the present study found the majority of disseminated cases of fusariosis (11/16) occurring in patients with acute lymphoblastic leukemia and acute myeloid leukemia. Disseminated fusariosis in these patients has a poor prognosis and mortality rates are close to 75% (Nucci and Anaissie, 2007; Campo et al., 2010). The treatment of this infection is a challenge and in the absence of better alternatives, voriconazole and amphotericin B are the most recommended therapies (Nucci and Anaissie, 2007; Nucci et al., 2014; Tortorano et al., 2014; Al-Hatmi et al., 2017).

Results from our sequence analysis show that twelve phylogenetic species within the *solani* complex were involved in 40 cases and responsible for 93% of the fusariosis in this study. In addition, three species were identified as belonging to the *fujikuroi* complex (7%). Of the 12 species and haplotypes of FSSC where the 40 strains were distributed, six belonged to previously described *Fusarium* species or varieties (*F. keratoplasticum, F. falciforme, F. petroliphilum, Fusarium solani sensu stricto, F. ambrosium*, and *F. solani* f. *xanthoxyli*), three to known haplotypes (FSSC 25, FSSC 35, FSSC 18, respectively), while two clades were recently described in *Neocosmospora* [FSSC 7 = *N. gamsii*, FSSC 20 = *N. suttoniana* (Figure 1)]; note that according to these authors (Sandoval-Denis and Crous, 2018) the entire *Fusarium solani* species complex phylogenetically constitutes a separate genus, *Neocosmospora*, but not all extant species have consistently been denominated, resulting in the use of two generic names for closely related species. One strain (Fu87) was identified as a novel phylogenetic lineage within FSSC and matched with LEMM 110739, which was previously reported by Guevara-Suarez et al. (2016) from an onychomycosis case. Numerous haplotypes and the newly reported lineage have remained yet unnamed. In the present study, *F. keratoplasticum* (FSSC 2) was the most often recorded species (28%), followed by *F. petroliphilum* (FSSC 1, 23.3%), which agrees with data of O’Donnell et al. (2007). In accordance with literature data (O’Donnell et al., 2007; Walther et al., 2017) we also encountered *Fusarium solani sensu stricto* (FSSC 5) causing keratitis.

Members of FSSC with a significant role in clinical infections in our data set comprised *F. falciforme* (FSSC 3+4), *F. keratoplasticum* (FSSC 2), *F. lichenicola* (FSSC 16), *F. metavorans* (FSSC 6), *F. petroliphilum* (FSSC 1), *F. pseudensiforme* (FSSC 33), and *F. solani sensu stricto* (FSSC 5) (Al-Hatmi et al., 2018a; Boral et al., 2018). Another lineage associated with opportunistic infections in FSSC that has been named is FSSC 27 (*Phialophora cyanescens* = *Cylindrocarpon cyanescens*), which was recently recombined as *Neocosmospora cyanescens*, MB 813864 (Summerbell and Scott, 2016). This species of FSSC lacks a name in *Fusarium*, while conversely *F. solani* f. *xanthoxyli* has no name in *Neocosmospora*; thus, consistent naming of the fungi in FSSC is impossible. Recently, a study from Japan also reported that haplotypes FSSC 9 and FSSC 18 are associated with opportunistic infections and with mycotic keratitis (Muraosa et al., 2017), while a German report found FSSC 9 and FSSC 25 to be involved in endophthalmitis (Walther et al., 2017). Literature data indicate that species within FSSC are the main cause of fusariosis worldwide (Scheel et al., 2013; Hassan et al., 2016; Tupaki-Sreepurna et al., 2017a). *Fusarium keratoplasticum* has been reported as the etiologic cause of disseminated fusariosis in hematologic patients (García-Ruiz et al., 2015; Chiewchanvit et al., 2017), as well as keratitis (Tupaki-Sreepurna et al., 2017a), onychomycoses (Guevara-Suarez et al., 2016; Gupta et al., 2016) and eumycetoma (Al-Hatmi et al., 2017). In addition, *F. keratoplasticum* is an important veterinary etiologic agent, causing disease in equine and marine vertebrates as well as in invertebrates (O’Donnell et al., 2016).

In the present study, we identified additional species and haplotypes for the first time from clinical samples, including *F. pseudensiforme* (FSSC 33), *F. solani* f. *xanthoxyli* (FSSC 22), *N. gamsii* (haplotype 7 – FSSC 7), *N. suttoniana* (haplotype 20 – FSSC 20), *Fusarium* sp. (FSSC 25), and *Fusarium* sp. (FSSC 35) (Figure 1), but confirmed case reports are as yet lacking. All these haplotypes are phylogenetically distinct from described species but remain unnamed as molecular siblings. Our data suggest that these additional species/haplotypes might be of importance for human health, although on the other hand it remains questionable whether formal description of the FSSC lineages as formal species is meaningful. Using *TEF1*α sequences strain Fu87 matched with an undescribed lineage (LEMM 110739) previously reported by Guevara-Suarez et al. (2016) from clinical samples in Colombia.

The number of reports of *Fusarium* species that were previously considered to be exclusive plant pathogens but are now implicated in superficial and systemic infections in humans and animals is obviously increasing (Zhang et al., 2006). *Fusarium* is rather unique in having pathogenic strategies to infect plants as well as animals including humans. This trans-kingdom pathogenicity has been demonstrated for the molecular siblings *F. falciforme, F. keratoplasticum* and *F. solani sensu stricto* within FSSC (Nalim et al., 2011; Short et al., 2013). Thus, our findings support the concept that *Fusarium* might serve as good model for studying the genetic basis of trans-kingdom pathogenicity in fungi (Ortoneda et al., 2004).

Our findings agree with reports from different regions in the world where the most frequently identified species causing human infections belonged to the FSSC followed by the *fujikuroi* and *oxysporum* species complexes (Al-Hatmi et al., 2015, 2016b; Taj-Aldeen et al., 2016). In Brazil species of FSSC were the most commonly reported, followed by the *fujikuroi* species complex (Scheel et al., 2013) and *oxysporum* species complex (Dallé da Rosa et al., 2018). Future studies including larger numbers of isolates are warranted to establish the prevalence of rare *Fusarium* species in clinical settings. In our study, *F. keratoplasticum* showed high MIC values (8–>32 μg mL^-1^) for most azoles tested and agricultural fungicides, with geometric mean MICs of 1.58 μg mL^-1^ for amphotericin B, 16 μg mL^-1^ for voriconazole and 64 μg mL^-1^ for posaconazole, the most effective drugs against *Fusarium* species (Lortholary et al., 2016). Rosa et al. (2017) observed that *F. keratoplasticum* was the species most frequently found in onychomycoses lesions and was more susceptible to amphotericin B and voriconazole than the other antifungals tested, with geometric mean MICs of 4.88 and 20.09 μg mL^-1^, respectively, higher than those observed in the present study. A study performed with 89 *Fusarium* isolates obtained from patients with superficial infections revealed that 49 (55.1%) of isolates belonged to *F. solani* species complex and 40 belonged to *F. oxysporum* species complex. Most of isolates showed high MIC values to antifungals tested, with modal MIC values of >16 μg mL^-1^ to amphotericin B, itraconazole, voriconazole, and posaconazole (Guevara-Suarez et al., 2016). Itraconazole had no *in vitro* effect against the isolates tested, which agrees with Tupaki-Sreepurna et al. (2017b). Similarly, Gupta et al. (2016) observed high MIC values of flucytosine, itraconazole, posaconazole, anidulafungin, and caspofungin for clinical isolates of *F. keratoplasticum*.

In view of the resistance of *Fusarium* spp. to several antifungal agents, some studies have tested its susceptibility to new antifungals. Abastabar et al. (2018) tested luliconazole, lanoconazole, and efinaconazole against clinical and environmental *Fusarium* isolates members of the *F. fujikuroi* species complex (*n* = 94), *F. solani* species complex (*n* = 14), *F. oxysporum* species complex (*n* = 11), *F. lateritium* species complex (*n* = 1), and *F. graminearum* species complex (*n* = 1). Overall, *Fusarium* species demonstrated lower MICs to luliconazole, lanoconazole and efinaconazole (geometric mean MICs of 0.005, 0.013, and 0.85 μg mL^-1^, respectively) when compared with voriconazole and amphotericin B (geometric mean MICs of 1.37 and 1.9 μg mL^-1^, respectively). In addition, Tupaki-Sreepurna et al. (2017b) tested the susceptibility of *F. solani* species complex (*n* = 18), *F. dimerum* species complex (*n* = 2), and *F. incarnatum-equiseti* species complex (*n* = 1) to efinaconazole. The concentrations of efinaconazole necessary to inhibited fungal growth vary from 0.031 to 2 μg mL^-1^, with geometric mean MICs varying from 0.08 to 0.7 μg mL^-1^ depending on *Fusarium* species. These data suggested that luliconazole, lanoconazole and efinaconazole are effective drugs that may be used against fusariosis.

## Conclusion

In conclusion, *F. keratoplasticum* and *F. petroliphilum* were the most frequent species in this study. Amphotericin B showed lower MICs against *Fusarium* species whereas the antifungal azoles and the fungicide difenoconazole exhibited higher MICs against FSSC.

## Ethics Statement

Samples were collected during routine patient care and the study was retrospective, therefore it was determined by the local Institutional Review Board of the Hospital de Clínicas, Federal University of Paraná and CAPES that ethical clearance was not indicated.

## Author Contributions

PH, AA-H, FQ-T, and JM designed the study. PH and AA-H performed the experiments and wrote the first draft. RP, MM, MN, FQ-T, GH, and JM analyzed the data and revised the manuscript. All authors contributed to the writing and approved the final manuscript.

## Conflict of Interest Statement

JM received grants from F2G and Merck. He has been a consultant to Scynexis and Merck and received speaker’s fees from Merck, United Medical, TEVA and Gilead Sciences. The remaining authors declare that the research was conducted in the absence of any commercial or financial relationships that could be construed as a potential conflict of interest.

## Footnotes

1 www.ebi.ac.uk/Tools/msa/mafft/

## References

[B1] Abastabar M. Al-Hatmi A. M. S. Vafaei Moghaddam M. de Hoog G. S. Haghani I. Aghili S. R. (2018). Potent activities of luliconazole, lanoconazole, and eight comparators against molecularly characterized *Fusarium* species. *Antimicrob. Agents Chemother.* 62:e00009–18. 10.1128/AAC.00009-18 29530844 PMC5923147

[B2] Al-Hatmi A. M. S. Bonifaz A. de Hoog G. S. Vazquez-Maya L. Garcia-Carmona K. Meis J. F. (2014). Keratitis by *Fusarium temperatum*, a novel opportunist. *BMC Infect. Dis.* 14:588. 10.1186/s12879-014-0588-y 25388601 PMC4234859

[B3] Al-Hatmi A. M. S. Ahmed S. A. van Diepeningen A. D. Drogari-Apiranthitou M. Verweij P. E. Meis J. F. (2018a). *Fusarium metavorans* sp. nov.: the frequent opportunist “FSSC6.”. *Med. Mycol.* 56 S144–S152. 10.1093/mmy/myx107 29538734

[B4] Al-Hatmi A. M. S. Bonifaz A. Ranque S. de Hoog G. S. Verweij P. E. Meis J. F. (2018b). Current antifungal treatment of fusariosis. *Int. J. Antimicrob. Agents* 51 326–332. 10.1016/j.ijantimicag.2017.06.017 28705676

[B5] Al-Hatmi A. M. S. Hagen F. Menken S. B. J. Meis J. F. de Hoog G. S. (2016a). Global molecular epidemiology and genetic diversity of *Fusarium*, a significant emerging human opportunist from 1958-2015. *Emerg. Microbes Infect.* 5:e124. 10.1038/emi.2016.126 27924809 PMC5180370

[B6] Al-Hatmi A. M. S. Meis J. F. de Hoog G. S. (2016b). *Fusarium*: molecular diversity and intrinsic drug resistance. *PLoS Pathog.* 12:e1005464. 10.1371/journal.ppat.1005464 27054821 PMC4824402

[B7] Al-Hatmi A. M. S. van Diepeningen A. D. Curfs-Breuker I. de Hoog G. S. Meis J. F. (2015). Specific antifungal susceptibility profiles of opportunists in the *Fusarium fujikuroi* complex. *J. Antimicrob. Chemother.* 70 1068–1071. 10.1093/jac/dku505 25538167

[B8] Alvarez-Moreno C. Lavergne R.-A. Hagen F. Morio F. Meis J. F. Le Pape P. (2017). Azole-resistant *Aspergillus fumigatus* harboring TR34/L98H, TR46/Y121F/T289A and TR53 mutations related to flower fields in Colombia. *Sci. Rep.* 7:45631. 10.1038/srep45631 28358115 PMC5372364

[B9] Al-Hatmi A. M. S. Bonifaz A. Tirado-Sánchez A. Meis J. F. de Hoog G. S. Ahmed S. A. (2017). *Fusarium* species causing eumycetoma: report of two cases and comprehensive review of the literature. *Mycoses* 60 204–212. 10.1111/myc.12590 27928841

[B10] Basler R. (2016). Diversity of *Fusarium* species isolated from UK forage maize and the population structure of *F. graminearum* from maize and wheat. *PeerJ* 4:e2143. 10.7717/peerj.2143 27366645 PMC4924121

[B11] Boral H. van Diepeningen A. Erdem E. Yağmur M. de Hoog G. S. Ilkit M. (2018). Mycotic keratitis caused by *Fusarium solani* sensu stricto (FSSC5): a case series. *Mycopathologia* 183 835–840. 10.1007/s11046-018-0280-7 29931660

[B12] Campo M. Lewis R. E. Kontoyiannis D. P. (2010). Invasive fusariosis in patients with hematologic malignancies at a cancer center: 1998–2009. *J. Infect.* 60 331–337. 10.1016/j.jinf.2010.01.010 20138081

[B13] Carlesse F. Amaral A.-P. C. Gonçalves S. S. Xafranski H. Lee M.-L. M. Zecchin V. (2017). Outbreak of *Fusarium oxysporum* infections in children with cancer: an experience with 7 episodes of catheter-related fungemia. *Antimicrob. Resist. Infect. Control* 6:93. 10.1186/s13756-017-0247-3 28912948 PMC5588724

[B14] Chiewchanvit S. Chongkae S. Mahanupab P. Nosanchuk J. D. Pornsuwan S. Vanittanakom N. (2017). Melanization of *Fusarium keratoplasticum* (*F. solani* species complex) during disseminated fusariosis in a patient with acute leukemia. *Mycopathologia* 182 879–885. 10.1007/s11046-017-0156-2 28616680

[B15] Chowdhary A. Kathuria S. Xu J. Meis J. F. (2013). Emergence of azole-resistant *Aspergillus fumigatus* strains due to agricultural azole use creates an increasing threat to human health. *PLoS Pathog.* 9:e1003633. 10.1371/journal.ppat.1003633 24204249 PMC3812019

[B16] Chowdhary A. Kathuria S. Xu J. Sharma C. Sundar G. Singh P. K. (2012). Clonal expansion and emergence of environmental multiple-triazole-resistant *Aspergillus fumigatus* strains carrying the TR34/L98H mutations in the cyp51A gene in India. *PLoS One* 7:e52871. 10.1371/journal.pone.0052871 23285210 PMC3532406

[B17] Clark N. Grim S. Lynch J. (2015). Posaconazole: use in the prophylaxis and treatment of fungal infections. *Semin. Respir. Crit. Care Med.* 36 767–785. 10.1055/s-0035-1562902 26398542

[B18] Clinical and Laboratory Standards Institute [CLSI] (2008). *Reference Method for Broth Dilution Antifungal Susceptibility Testing of Filamentous Fungi; Approved Standard.* Wayne, PA: CLSI.

[B19] Costa S. S. Matos K. S. Tessmann D. J. Seixas C. D. S. Pfenning L. H. (2016). *Fusarium paranaense* sp. nov., a member of the *Fusarium solani* species complex causes root rot on soybean in Brazil. *Fungal Biol.* 120 51–60. 10.1016/j.funbio.2015.09.005 26693684

[B20] Dallé da Rosa P. Nunes A. Borges R. Batista B. Meneghello Fuentefria A. Goldani L. Z. (2018). In vitro susceptibility and multilocus sequence typing of *Fusarium* isolates causing keratitis. *J. Mycol. Med.* 28 482–485. 10.1016/j.mycmed.2018.05.001 29779647

[B21] de Souza M. Matsuzawa T. Lyra L. Busso-Lopes A. Gonoi T. Schreiber A. (2014). *Fusarium napiforme* systemic infection: case report with molecular characterization and antifungal susceptibility tests. *SpringerPlus* 3:492. 10.1186/2193-1801-3-492 25210666 PMC4159480

[B22] Deising H. B. Reimann S. Pascholati S. F. (2008). Mechanisms and significance of fungicide resistance. *Braz. J. Microbiol.* 39 286–295. 10.1590/S1517-838220080002000017 24031218 PMC3768401

[B23] Espinel-Ingroff A. Colombo A. L. Cordoba S. Dufresne P. J. Fuller J. Ghannoum M. (2016). International evaluation of MIC distributions and epidemiological cut-off value (ECV) definitions for *Fusarium* species identified by molecular methods for the CLSI broth microdilution method. *Antimicrob. Agents Chemother.* 60 1079–1084. 10.1128/AAC.02456-15 26643334 PMC4750715

[B24] Fan J. Urban M. Parker J. E. Brewer H. C. Kelly S. L. Hammond-Kosack K. E. (2013). Characterization of the sterol 14α-demethylases of *Fusarium graminearum* identifies a novel genus-specific CYP51 function. *New Phytol.* 198 821–835. 10.1111/nph.12193 23442154

[B25] García-Ruiz J. C. Olazábal I. Adán Pedroso R. M. López-Soria L. Velasco-Benito V. Sánchez-Aparicio J. A. (2015). Disseminated fusariosis and hematologic malignancies, a still devastating association. Report of three new cases. *Rev. Iberoam. Micol.* 32 190–196. 10.1016/j.riam.2014.11.003 25936697

[B26] Gauthier G. M. Keller N. P. (2013). Crossover fungal pathogens: the biology and pathogenesis of fungi capable of crossing kingdoms to infect plants and humans. *Fungal Genet. Biol.* 61 146–157. 10.1016/j.fgb.2013.08.016 24021881

[B27] Geiser D. M. Jimenez-Gasco M. D. Kang S. C. Makalowska I. Veeraraghavan N. Ward T. J. (2004). FUSARIUM-ID v.1.0: a DNA sequence database for identifying *Fusarium*. *Eur. J. Plant Pathol.* 110 473–479.

[B28] Guevara-Suarez M. Cano-Lira J. F. Cepero de García M. C. Sopo L. De Bedout C. Cano L. E. (2016). Genotyping of *Fusarium* isolates from onychomycoses in Colombia: detection of two new species within the *Fusarium solani* species complex and in vitro antifungal susceptibility testing. *Mycopathologia* 181 165–174. 10.1007/s11046-016-9983-9 26943726

[B29] Gupta C. Jongman M. Das S. Snehaa K. Bhattacharya S. N. Seyedmousavi S. (2016). Genotyping and in vitro antifungal susceptibility testing of *Fusarium* isolates from onychomycosis in India. *Mycopathologia* 181 497–504. 10.1007/s11046-016-0014-7 27138574

[B30] Hassan A. S. Al-Hatmi A. M. S. Shobana C. S. van Diepeningen A. D. Kredics L. Vágvölgyi C. (2016). Antifungal susceptibility and phylogeny of opportunistic members of the genus *Fusarium* causing human keratomycosis in South India. *Med. Mycol.* 54 287–294. 10.1093/mmy/myv105 26705832

[B31] Hof H. (2001). Critical annotations to the use of azole antifungals for plant protection. *Antimicrob. Agents Chemother.* 45 2987–2990. 10.1128/AAC.45.11.2987-2990.2001 11600346 PMC90772

[B32] Kassar O. Charfi M. Trabelsi H. Hammami R. Elloumi M. (2016). *Fusarium solani* endocarditis in an acute leukemia patient. *Med. Mal. Infect.* 46 57–59. 10.1016/j.medmal.2015.11.004 26706407

[B33] Katiyar S. K. Edlind T. D. (2009). Role for Fks1 in the intrinsic echinocandin resistance of *Fusarium solani* as evidenced by hybrid expression in *Saccharomyces cerevisiae*. *Antimicrob. Agents Chemother.* 53 1772–1778. 10.1128/AAC.00020-09 19258277 PMC2681557

[B34] Khodavaisy S. Badali H. Rezaie S. Nabili M. Moghadam K. G. Afhami S. (2016). Genotyping of clinical and environmental *Aspergillus flavus* isolates from Iran using microsatellites. *Mycoses* 59 220–225. 10.1111/myc.12451 26756650

[B35] Kim S. W. Park J. K. Lee C. H. Hahn B.-S. Koo J. C. (2016). Comparison of the antimicrobial properties of chitosan oligosaccharides (COS) and EDTA against *Fusarium fujikuroi* causing rice bakanae disease. *Curr. Microbiol.* 72 496–502. 10.1007/s00284-015-0973-9 26729353

[B36] Lortholary O. Fernández-Ruiz M. Perfect J. R. (2016). The current treatment landscape: other fungal diseases (cryptococcosis, fusariosis and mucormycosis). *J. Antimicrob. Chemother.* 71 ii31–ii36. 27880667 10.1093/jac/dkw394

[B37] Lortholary O. Obenga G. Biswas P. Caillot D. Chachaty E. Bienvenu A.-L. (2010). International retrospective analysis of 73 cases of invasive fusariosis treated with voriconazole. *Antimicrob. Agents Chemother.* 54 4446–4450. 10.1128/AAC.00286-10 20625156 PMC2944599

[B38] Manzo D. Ferriello F. Puopolo G. Zoina A. D’Esposito D. Tardella L. (2016). *Fusarium oxysporum* f.sp. Radicis-lycopersici induces distinct transcriptome reprogramming in resistant and susceptible isogenic tomato lines. *BMC Plant Biol.* 16:53. 10.1186/s12870-016-0740-5 26920134 PMC4769521

[B39] Meis J. F. Chowdhary A. Rhodes J. L. Fisher M. C. Verweij P. E. (2016). Clinical implications of globally emerging azole resistance in *Aspergillus fumigatus*. *Philos. Trans. R. Soc. Lond. B. Biol. Sci.* 371:20150460. 10.1098/rstb.2015.0460 28080986 PMC5095539

[B40] Milicevic D. R. Skrinjar M. Baltic T. (2010). Real and perceived risks for mycotoxin contamination in foods and feeds: challenges for food safety control. *Toxins* 2 572–592. 10.3390/toxins2040572 22069600 PMC3153222

[B41] Muraosa Y. Oguchi M. Yahiro M. Watanabe A. Yaguchi T. Kamei K. (2017). Epidemiological study of *Fusarium* species causing invasive and superficial fusariosis in Japan. *Med. Mycol. J.* 58 E5–E13. 10.3314/mmj.16-00024 28250364

[B42] Nalim F. A. Samuels G. J. Wijesundera R. L. Geiser D. M. (2011). New species from the *Fusarium solani* species complex derived from perithecia and soil in the Old World tropics. *Mycologia* 103 1302–1330. 10.3852/10-307 21700636

[B43] Nucci F. Nouer S. Capone D. Anaissie E. Nucci M. (2015). Fusariosis. *Semin. Respir. Crit. Care Med.* 36 706–714. 10.1055/s-0035-1562897 26398537

[B44] Nucci M. Anaissie E. (2007). *Fusarium* infections in immunocompromised patients. *Clin. Microbiol. Rev.* 20 695–704. 10.1128/CMR.00014-07 17934079 PMC2176050

[B45] Nucci M. Anaissie E. J. Queiroz-Telles F. Martins C. A. Trabasso P. Solza C. (2003). Outcome predictors of 84 patients with hematologic malignancies and *Fusarium* infection: *Fusarium* prognostic factors. *Cancer* 98 315–319. 10.1002/cncr.11510 12872351

[B46] Nucci M. Marr K. A. Queiroz-Telles F. Martins C. A. Trabasso P. Costa S. (2004). *Fusarium* infection in hematopoietic stem cell transplant recipients. *Clin. Infect. Dis.* 38 1237–1242. 10.1086/383319 15127334

[B47] Nucci M. Marr K. A. Vehreschild M. J. G. T. de Souza C. A. Velasco E. Cappellano P. (2014). Improvement in the outcome of invasive fusariosis in the last decade. *Clin. Microbiol. Infect.* 20 580–585. 10.1111/1469-0691.12409 24118322

[B48] Nucci M. Shoham S. Abdala E. Hamerschlak N. Rico J. C. Forghieri F. (2019). Outcomes of patients with invasive fusariosis who undergo further immunosuppressive treatments, is there a role for secondary prophylaxis? *Mycoses* 10.1111/myc.12901 [Epub ahead of print]. 30720902

[B49] Nucci M. Varon A. G. Garnica M. Akiti T. Barreiros G. Trope B. M. (2013). Increased incidence of invasive fusariosis with cutaneous portal of entry, Brazil. *Emerg. Infect. Dis.* 19 1567–1572. 10.3201/eid1910.120847 24050318 PMC3810727

[B50] O’Donnell K. Kistler H. C. Cigelnik E. Ploetz R. C. (1998). Multiple evolutionary origins of the fungus causing Panama disease of banana: concordant evidence from nuclear and mitochondrial gene genealogies. *Proc. Natl. Acad. Sci. U.S.A.* 95 2044–2049. 9482835 10.1073/pnas.95.5.2044 PMC19243

[B51] O’Donnell K. Sarver B. A. Brandt M. Chang D. C. Noble-Wang J. Park B. J. (2007). Phylogenetic diversity and microsphere array-based genotyping of human pathogenic Fusaria, including isolates from the multistate contact lens-associated U.S. keratitis outbreaks of 2005 and 2006. *J. Clin. Microbiol.* 45 2235–2248. 17507522 10.1128/JCM.00533-07 PMC1933018

[B52] O’Donnell K. Sutton D. A. Rinaldi M. G. Sarver B. A. Balajee S. A. Schroers H. J. (2010). Internet-accessible DNA sequence database for identifying fusaria from human and animal infections. *J. Clin. Microbiol.* 48 3708–3718. 10.1128/JCM.00989-10 20686083 PMC2953079

[B53] O’Donnell K. Sutton D. A. Wiederhold N. Robert V. A. R. G. Crous P. W. Geiser D. M. (2016). Veterinary fusarioses within the United States. *J. Clin. Microbiol.* 54 2813–2819. 10.1128/JCM.01607-16 27605713 PMC5078561

[B54] Ortoneda M. Guarro J. Madrid M. P. Caracuel Z. Roncero M. I. G. Mayayo E. (2004). *Fusarium oxysporum* as a multihost model for the genetic dissection of fungal virulence in plants and mammals. *Infect. Immun.* 72 1760–1766. 10.1128/IAI.72.3.1760-1766.2004 14977985 PMC356063

[B55] Rosa P. D. Heidrich D. Corrêa C. Scroferneker M. L. Vettorato G. Fuentefria A. M. (2017). Genetic diversity and antifungal susceptibility of *Fusarium* isolates in onychomycosis. *Mycoses* 60 616–622. 10.1111/myc.12638 28657120

[B56] Salah H. Al-Hatmi A. M. Theelen B. Abukamar M. Hashim S. van Diepeningen A. D. (2015). Phylogenetic diversity of human pathogenic *Fusarium* and emergence of uncommon virulent species. *J. Infect.* 71 658–666. 10.1016/j.jinf.2015.08.011 26348828

[B57] Sandoval-Denis M. Crous P. W. (2018). Removing chaos from confusion: assigning names to common human and animal pathogens in *Neocosmospora*. *Persoonia* 41 109–129. 10.3767/persoonia.2018.41.06 30728601 PMC6344815

[B58] Scheel C. M. Hurst S. F. Barreiros G. Akiti T. Nucci M. Balajee S. A. (2013). Molecular analyses of *Fusarium* isolates recovered from a cluster of invasive mold infections in a Brazilian hospital. *BMC Infect. Dis.* 13:49. 10.1186/1471-2334-13-49 23363475 PMC3579725

[B59] Short D. P. G. O’Donnell K. Thrane U. Nielsen K. F. Zhang N. Juba J. H. (2013). Phylogenetic relationships among members of the *Fusarium solani* species complex in human infections and the descriptions of *F. keratoplasticum* sp. nov. and *F. petroliphilum* stat. nov. *Fungal Genet. Biol.* 53 59–70. 10.1016/j.fgb.2013.01.004 23396261

[B60] Sierra-Hoffman M. Paltiyevich-Gibson S. Carpenter J. L. Hurley D. L. (2005). *Fusarium* osteomyelitis: case report and review of the literature. *Scand. J. Infect. Dis.* 37 237–240. 10.1080/00365540410021036 15849061

[B61] Singh R. P. Singh P. K. Rutkoski J. Hodson D. P. He X. Jørgensen L. N. (2016). Disease impact on wheat yield potential and prospects of genetic control. *Annu. Rev. Phytopathol.* 54 303–322. 10.1146/annurev-phyto-080615-095835 27296137

[B62] Snelders E. van der Lee H. A. L. Kuijpers J. Rijs A. J. M. M. Varga J. Samson R. A. (2008). Emergence of azole resistance in *Aspergillus fumigatus* and spread of a single resistance mechanism. *PLoS Med.* 5:e219. 10.1371/journal.pmed.0050219 18998768 PMC2581623

[B63] Sobrova P. Adam V. Vasatkova A. Beklova M. Zeman L. Kizek R. (2010). Deoxynivalenol and its toxicity. *Interdiscip. Toxicol.* 3 94–99. 10.2478/v10102-010-0019-x 21217881 PMC2984136

[B64] Su C. Hsu H. Wu J. Chou C. (2007). Diagnosis of fusariosis in urine cytology. *J. Clin. Pathol.* 60 422–424. 10.1136/jcp.2006.038489 16816172 PMC2001109

[B65] Summerbell R. C. Scott J. A. (2016). “Conidiogenesis: Its evolutionary aspects in the context of a philosophy of opportunity (lectics),” in *Biology of Microfungi Fungal Biology*, ed. Li D.-W. (Cham: Springer International Publishing), 169–195. 10.1007/978-3-319-29137-6-8

[B66] Taj-Aldeen S. J. Salah H. Al-Hatmi A. M. Hamed M. Theelen B. van Diepeningen A. D. (2016). In vitro resistance of clinical *Fusarium* species to amphotericin B and voriconazole using the EUCAST antifungal susceptibility method. *Diagn. Microbiol. Infect. Dis.* 85 438–443. 10.1016/j.diagmicrobio.2016.05.006 27312690

[B67] Tamura K. Stecher G. Peterson D. Filipski A. Kumar S. (2013). MEGA6: molecular evolutionary genetics analysis version 6.0. *Mol. Biol. Evol.* 30 2725–2729. 10.1093/molbev/mst197 24132122 PMC3840312

[B68] Tortorano A. M. Richardson M. Roilides E. Diepeningen A. van Caira M. Munoz P. (2014). ESCMID and ECMM joint guidelines on diagnosis and management of hyalohyphomycosis: *Fusarium* spp., *Scedosporium* spp. and others. *Clin. Microbiol. Infect.* 20 27–46. 10.1111/1469-0691.12465 24548001

[B69] Tupaki-Sreepurna A. Al-Hatmi A. M. S. Kindo A. J. Sundaram M. de Hoog G. S. (2017a). Multidrug-resistant *Fusarium* in keratitis: a clinico-mycological study of keratitis infections in Chennai, India. *Mycoses* 60 230–233. 10.1111/myc.12578 27766684

[B70] Tupaki-Sreepurna A. Jishnu B. T. Thanneru V. Sharma S. Gopi A. Sundaram M. (2017b). An assessment of in vitro antifungal activities of efinaconazole and itraconazole against common non-dermatophyte fungi causing onychomycosis. *J. Fungi* 3:20. 10.3390/jof3020020 29371538 PMC5715924

[B71] van Diepeningen A. D. Al-Hatmi A. M. S. Brankovics B. de Hoog G. S. (2014). Taxonomy and clinical spectra of *Fusarium* species: where do we stand in 2014? *Curr. Clin. Microbiol. Rep.* 1 10–18. 10.1007/s40588-014-0003-x

[B72] van Diepeningen A. D. de Hoog G. S. (2016). Challenges in *Fusarium*, a trans-kingdom pathogen. *Mycopathologia* 181 161–163. 10.1007/s11046-016-9993-7 26966007

[B73] Verweij P. E. Chowdhary A. Melchers W. J. G. Meis J. F. (2016). Azole resistance in *Aspergillus fumigatus*: can we retain the clinical use of mold-active antifungal azoles? *Clin. Infect. Dis.* 62 362–368. 10.1093/cid/civ885 26486705 PMC4706635

[B74] Walther G. Stasch S. Kaerger K. Hamprecht A. Roth M. Cornely O. A. (2017). *Fusarium* keratitis in Germany. *J. Clin. Microbiol.* 55 2983–2995. 10.1128/JCM.00649-17 28747368 PMC5625384

[B75] Zhang N. O’Donnell K. Sutton D. A. Nalim F. A. Summerbell R. C. Padhye A. A. (2006). Members of the *Fusarium solani* species complex that cause infections in both humans and plants are common in the environment. *J. Clin. Microbiol.* 44 2186–2190. 10.1128/JCM.00120-06 16757619 PMC1489407

